# Differences in prevalence and predictive value of metabolic syndrome: A comparative study of IDF, CDS, and NCEP ATP III criteria in young Chinese healthcare workers

**DOI:** 10.1097/MD.0000000000050693

**Published:** 2026-10-02

**Authors:** Li Ma, Lei Lei, Zimin Cheng, Yuanyuan Lv, Jie Zheng, Xinyu Chen, Yimeng Huang, Xiaolei Luo, Xiaoliu Li

**Affiliations:** a Department of Cardiovascular Internal Medicine, Tianyou Hospital Affiliated to Wuhan University of Science and Technology, Wuhan, Hubei, China; b Department of Health Examination and Health Care, Tianyou Hospital Affiliated to Wuhan University of Science and Technology, Wuhan, Hubei, China; c Department of Rehabilitation, Minhang Hospital, Fudan University, Shanghai, China.

**Keywords:** cardiovascular risk, China-PAR, Chinese Diabetes Society, healthcare workers, International Diabetes Federation, metabolic syndrome, NCEP ATP III

## Abstract

Metabolic syndrome (MetS) is common in Chinese adults, but evidence regarding its burden and cardiovascular relevance among young healthcare workers (HCWs) remains limited. Different MetS definitions can yield materially different prevalence estimates and risk classifications. This retrospective cross-sectional study included 420 HCWs aged 20 to 45 years who underwent mandatory annual health examinations at a tertiary hospital in China from 2022 to 2024. Demographic, occupational, anthropometric, and biochemical data were extracted from health examination and personnel records. MetS was defined using the International Diabetes Federation (IDF), revised National Cholesterol Education Program Adult Treatment Panel III (NCEP ATP III), and Chinese Diabetes Society (CDS) criteria. Ten-year atherosclerotic cardiovascular disease risk was estimated using the China-PAR equations, with elevated estimated risk defined as ≥10%. We compared MetS prevalence, sex differences, agreement among definitions, diagnostic performance using NCEP ATP III. Participants had a mean age of 32.4 ± 5.8 years, and 30.0% were men. MetS prevalence was 15.5%, 18.6%, and 10.2% according to IDF, NCEP ATP III, and CDS criteria, respectively, and was consistently higher in men. Agreement was almost perfect between IDF and NCEP ATP III (κ = 0.82) and substantial between IDF and CDS (κ = 0.64) and between NCEP ATP III and CDS (κ = 0.61). Using an internally reconstructed 2 × 2 table that preserves the reported prevalence counts and pairwise κ values, IDF showed sensitivity 78.2%, specificity 98.8%, PPV 93.8%, and NPV 95.2%, whereas CDS showed sensitivity 51.3%, specificity 99.1%, PPV 93.0%, and NPV 89.9% relative to NCEP ATP III. These reconstructed diagnostic-performance values require verification against the original participant-level data before submission. MetS defined by any criterion was strongly associated with elevated China-PAR-estimated risk, with adjusted odds ratio of 6.5, 7.1, and 5.1 for IDF, NCEP ATP III, and CDS, respectively. atherosclerotic cardiovascular disease-risk AUCs were 0.80, 0.82, and 0.76, respectively. In this single-center cohort of young Chinese HCWs, estimated MetS prevalence differed substantially by diagnostic definition. IDF and NCEP ATP III identified more cases than CDS and showed stronger mutual agreement. The observed associations with China-PAR-estimated risk support the potential value of MetS assessment within occupational health screening.

## 1. Introduction

Metabolic syndrome (MetS) is a clustering of central obesity, dyslipidaemia, elevated blood pressure, and impaired glucose regulation that is associated with increased risks of type 2 diabetes and cardiovascular disease. The International Diabetes Federation (IDF) proposed a worldwide definition in 2005 that emphasizes central obesity and ethnic-specific waist-circumference cutoffs.^[1]^ A systematic review and meta-analysis reported that MetS is associated with an approximately 2-fold higher risk of cardiovascular events and increased all-cause mortality.^[2]^

Several expert groups have proposed diagnostic criteria for MetS. The revised National Cholesterol Education Program Adult Treatment Panel III (NCEP ATP III) definition treats the 5 components as equally weighted, whereas the IDF requires central obesity. The 2004 Chinese Diabetes Society (CDS) criteria were adapted to Chinese clinical practice and use different thresholds, including body mass index (BMI) rather than waist circumference (WC) for the obesity component.^[3,4]^ Consequently, the same individual may be classified differently depending on the definition used.

Large Chinese studies have repeatedly shown that definition choice materially changes MetS prevalence. Earlier national and regional studies generally reported higher prevalence using revised ATP III than using IDF or CDS criteria.^[5-8]^ More recently, Ma et al compared 5 definitions in 9588 Chinese adults and again found substantial variation in prevalence across definitions, reinforcing the need to interpret MetS estimates in the context of the diagnostic system used.

Working populations deserve particular attention because long sedentary periods, shift work, psychosocial stress, sleep disruption, and limited opportunities for regular exercise may contribute to cardiometabolic risk. In healthcare settings, Taiwanese studies have reported MetS prevalence and risk-factor patterns that vary by profession and sex.^[9-13]^ A 2024 study of 1110 healthcare workers (HCWs) in a Chinese tertiary hospital further demonstrated a high burden of cardiovascular-kidney-metabolic abnormalities, especially among men. In 2026, 2 large occupational health examination studies in China reported substantial MetS burdens among university employees and civil servants, with pronounced sex differences and potentially modifiable lifestyle correlates.

Despite this evidence, direct comparisons of IDF, CDS, and revised NCEP ATP III criteria in young Chinese HCWs remain limited. This study therefore aimed to compare prevalence, sex differences, agreement, and cardiovascular-risk discrimination across the 3 definitions in young HCWs. We specifically sought to determine whether definition choice meaningfully changes occupational screening classification and whether MetS status is associated with elevated Prediction for Atherosclerotic Cardiovascular Disease Risk in China (China-PAR)-estimated atherosclerotic cardiovascular disease (ASCVD) risk.

## 2. Materials and methods

### 2.1. Study design and participants

This study was approved by the Ethics Committee of Minhang Hospital, Fudan University. This retrospective cross-sectional study was conducted among young HCWs at a single tertiary hospital in China. Data were obtained from the hospital’s occupational health examination database and personnel records. All HCWs aged 20 to 45 years who underwent routine annual health examinations between January 2022 and December 2024 were screened for eligibility. The annual health examination is mandatory for all employees and includes a standardized questionnaire, physical examination and laboratory tests. To avoid correlated observations from repeated annual examinations, each employee contributed only 1 record to the final cross-sectional dataset. When more than 1 examination was available during 2022 to 2024, the most recent eligible examination with complete data was retained, and earlier duplicate records were excluded before statistical analysis.

Inclusion criteria were: age 20 to 45 years at the time of the retained examination; current employment as a physician, nurse, medical technician, administrative or logistic staff; and complete data on WC, blood pressure, fasting plasma glucose (FPG), triglycerides (TG), high-density lipoprotein cholesterol (HDL-C), total cholesterol, smoking status, diabetes status and other variables required for MetS classification and China-PAR risk estimation.

Exclusion criteria were: pregnancy; history of malignant tumor, severe hepatic or renal insufficiency, or other serious systemic disease; missing data on any component required for the definitions of MetS or for China-PAR risk estimation; and known history of myocardial infarction, stroke or other ASCVD at baseline. The relevant confidence data is incomplete or missing.

During the study period, 548 annual health examination records from HCWs aged 20 to 45 years were initially retrieved. Because some employees underwent more than 1 annual examination between 2022 and 2024, 72 duplicate examination records were removed by retaining only the most recent eligible examination with complete data for each employee. This resulted in 476 unique HCWs assessed for eligibility. A further 56 participants were excluded because of pregnancy (n = 8), serious systemic disease (n = 5), missing variables required for MetS classification or China-PAR estimation (n = 27), previous ASCVD (n = 3), or incomplete or internally inconsistent information that could not be resolved after source verification (n = 13). Consequently, 420 participants were included in the final analysis (Fig. 1).

**Figure 1.**
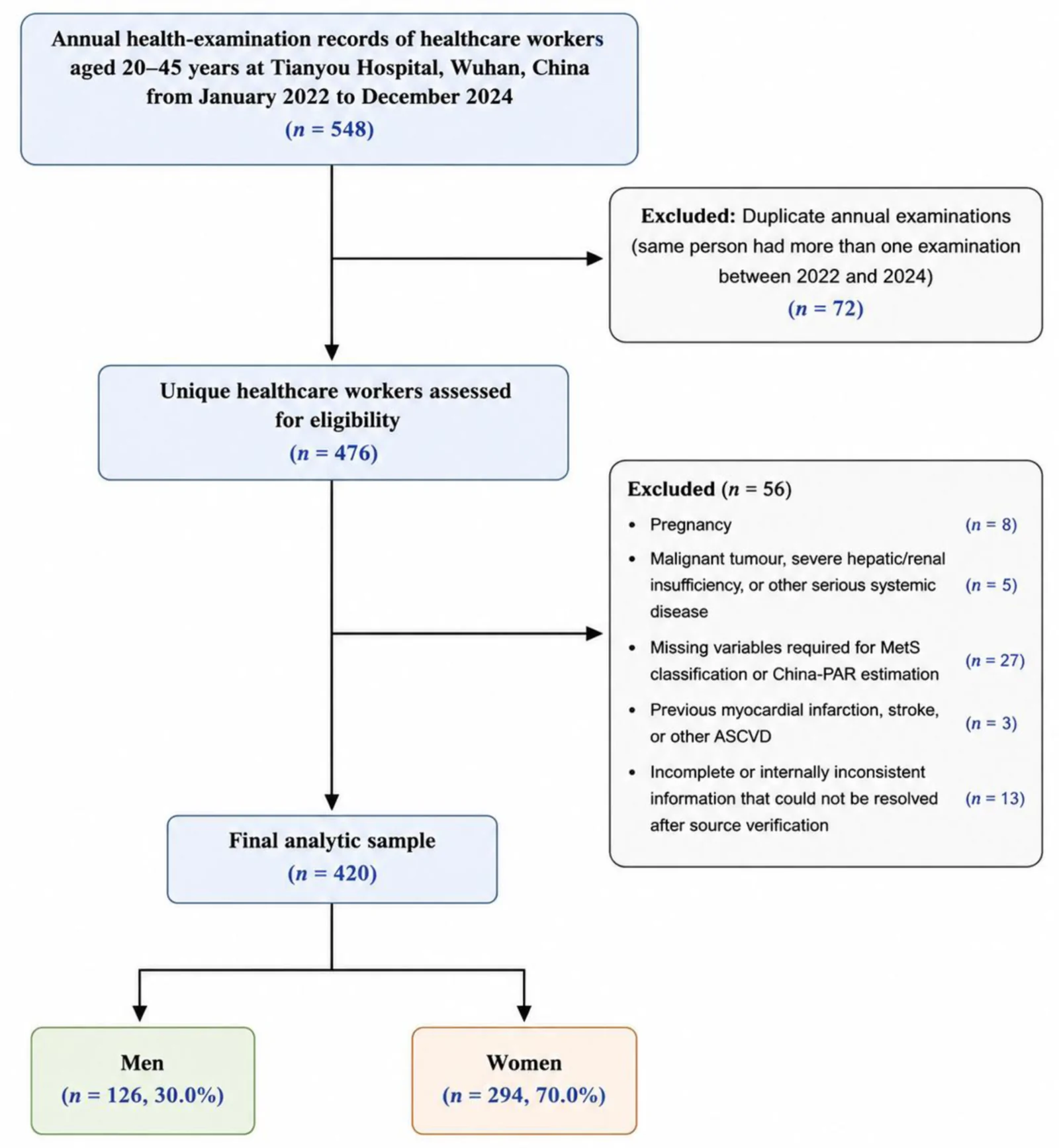
Flowchart of study population selection. ASCVD = atherosclerotic cardiovascular disease.

Data quality was checked before analysis. Records with internally inconsistent demographic or examination information were verified against the original occupational health examination report and, when applicable, electronic medical records. Extreme or physiologically implausible values for anthropometric, blood pressure or biochemical measurements were rechecked against source records; The final analyses used a complete-case approach, and no statistical imputation was performed.

After applying these criteria, a total of 420 young HCWs were included in the final analysis. The study protocol was approved by the Institutional Review Board. As only de-identified data from existing records were used, the requirement for written informed consent was waived.

### 2.2. Data collection

Demographic and occupational information, including age, sex, job category (physician, nurse, technician, administrative staff, other) and duration of employment, was retrieved from the hospital personnel system and linked to health examination records. Lifestyle factors were obtained from the standardized questionnaire routinely completed before the examination. Smoking status was classified as never, former or current smoker. Alcohol consumption was categorized as nondrinker, occasional drinker or regular drinker. Self-reported regular physical activity was grouped into low, moderate or high according to the frequency and duration of leisure-time exercise. Average nocturnal sleep duration was recorded in hours and further categorized as <6 h, 6–8 hours or >8 hours. Use of antihypertensive, lipid-lowering and antidiabetic medications, as well as previous diagnoses of hypertension and diabetes, was assessed by questionnaire and, when possible, cross-checked against electronic medical records.

### 2.3. Anthropometric and blood pressure measurements

All physical examinations were performed by trained nurses following standardized procedures. Body weight and height were measured with participants wearing light clothing and no shoes, using calibrated electronic scales and stadiometers, and recorded to the nearest 0.1 kg and 0.1 cm, respectively. BMI was calculated as weight (kg) divided by height squared (m^2^). WC was measured to the nearest 0.1 cm at the midpoint between the lower margin of the rib cage and the iliac crest, with participants standing, abdomen relaxed, and at the end of a normal expiration. Seated blood pressure was measured on the right arm using an automated sphygmomanometer (HEM-907, Omron Healthcare Co., Kyoto, Japan) after at least 5 minutes of rest. An appropriate cuff size was selected according to mid-arm circumference. Three measurements were taken at 1 to 2 minute intervals, and the mean of the 3 readings was used for analysis. Systolic blood pressure (SBP) and diastolic blood pressure (DBP) were expressed in millimeters of mercury (mm Hg).

### 2.4. Laboratory measurements

After an overnight fast of at least 10 to 12 hours, venous blood samples were drawn in the morning. FPG, TG, HDL-C, and total cholesterol were measured using an automated biochemical analyzer (Cobas 8000 modular analyzer series, Roche Diagnostics, Mannheim, Germany) in the central laboratory. All assays were performed with reagents from the same manufacturer (Roche Diagnostics) according to the manufacturer’s instructions. Internal quality control procedures were implemented throughout the study period, including the use of commercial control materials and regular calibration of instruments, in accordance with the laboratory’s standard operating protocols.

### 2.5. Definitions of metabolic syndrome

MetS was defined according to 3 sets of diagnostic criteria: the IDF, the revised NCEP ATP III, and the CDS.

#### 2.5.1. IDF (2005) definition

MetS was diagnosed if central obesity (required) plus at least two of the following components were present:

1. Central obesity: WC ≥ 90 cm in men or ≥80 cm in women (Asian cutoffs);
2. Raised TG: TG ≥ 1.7 mmol/L, or specific treatment for elevated TG;
3. Reduced HDL-C: <1.03 mmol/L in men or <1.29 mmol/L in women, or specific treatment for reduced HDL-C;
4. Raised blood pressure: SBP ≥ 130 mm Hg or DBP ≥ 85 mm Hg, or current use of antihypertensive medication;
5. Raised fasting glucose: FPG ≥ 5.6 mmol/L or previously diagnosed type 2 diabetes.

#### 2.5.2. Revised NCEP ATP III definition

MetS was diagnosed if at least 3 of the following 5 components were present:

1. Abdominal obesity: WC ≥ 90 cm in men or ≥80 cm in women (Asian cutoffs);
2. Raised TG: TG ≥ 1.7 mmol/L, or specific treatment for elevated TG;
3. Reduced HDL-C: <1.03 mmol/L in men or <1.29 mmol/L in women, or specific treatment for reduced HDL-C;
4. Raised blood pressure: SBP ≥ 130 mm Hg or DBP ≥ 85 mm Hg, or current use of antihypertensive medication;
5. Raised fasting glucose: FPG ≥ 5.6 mmol/L or previously diagnosed diabetes.

#### 2.5.3. CDS (2004) definition

According to the CDS, MetS was diagnosed when 3 or more of the following 5 abnormalities were present:

1. Overweight or obesity: BMI ≥ 25 kg/m^2^;
2. Hyperglycemia: FPG ≥ 6.1 mmol/L and/or 2h-PG ≥ 7.8 mmol/L, or previously diagnosed diabetes;
3. Elevated blood pressure: SBP ≥ 140 mm Hg and/or DBP ≥ 90 mm Hg, or current use of antihypertensive medication;
4. Raised TG: TG ≥ 1.7 mmol/L;
5. Reduced HDL-C: <0.9 mmol/L in men or <1.0 mmol/L in women.

### 2.6. Estimation of 10-year cardiovascular risk

Ten-year risk of ASCVD was estimated for each participant using the China-PAR equations, which were developed and validated in large contemporary Chinese cohorts. The China-PAR model incorporates age, sex, SBP, total cholesterol, HDL-C, smoking status, and presence of diabetes to calculate the absolute 10-year risk of first ASCVD events.^[11]^ Based on current Chinese recommendations, participants were categorized into low- and elevated-risk groups according to their estimated 10-year ASCVD risk. For the main analyses, elevated cardiovascular risk was defined as a China-PAR-estimated 10-year ASCVD risk ≥ 10%, and we examined the ability of each MetS definition to identify participants in this elevated-risk category. The China-PAR equations were originally developed in adults aged 35 to 74 years; therefore, application to participants aged < 35 years in the present study represents an extrapolation beyond the original derivation and validation age range. Among all participants, 344 (81.9%) were younger than 35 years, and 76 (18.1%) were aged 35 to 45 years. Results based on the full sample should therefore be interpreted as estimated risk stratification rather than formal long-term risk prediction for participants below the validated age range. Because the subgroup within the validated age range was relatively small, any age-restricted analysis was considered exploratory and was not used as the primary basis for inference.

### 2.7. Statistical analysis

Continuous variables were summarized as means ± standard deviations or medians (interquartile ranges), and categorical variables as counts and percentages. Group comparisons were performed using appropriate parametric or nonparametric tests for continuous variables and χ^2^ or Fisher’s exact tests for categorical variables. The prevalence of MetS according to each definition (IDF, NCEP ATP III, and CDS) was calculated with 95% confidence intervals (CIs), and pairwise differences were compared using McNemar’s test. Agreement between definitions was assessed using Cohen’s kappa (κ). NCEP ATP III was used as a pragmatic reference definition for sensitivity, specificity and Youden index calculations because it is widely used in epidemiological and occupational screening studies, treats the 5 MetS components as equally weighted, applies Asian waist-circumference cutoffs in the revised version, and identified the broadest group of MetS cases in this cohort. This choice was intended to facilitate comparative screening-performance analyses and should not be interpreted as implying that NCEP ATP III is a true diagnostic gold standard. The association between MetS and elevated 10-year cardiovascular risk (China-PAR-estimated ASCVD risk ≥ 10%) was evaluated using multivariable logistic regression, and the discriminative ability of the 3 definitions was compared by receiver operating characteristic (ROC) curves and area under the curves (AUCs). Pairwise comparisons of AUCs were performed using the nonparametric DeLong test. Because China-PAR was developed for adults aged 35 to 74 years, sensitivity analyses restricted to participants aged 35 to 45 years were also conducted to assess the robustness of the findings within the validated age range. All analyses were performed using IBM Statistical Package for the Social Sciences (SPSS) Statistics, version 26.0 (IBM Corp., Armonk), with a 2-sided *P* < .05 considered statistically significant.

## 3. Results

### 3.1. Baseline characteristics by sex

Among the 420 HCWs included in the analysis, 126 (30.0%) were men and 294 (70.0%) were women. The overall mean age was 32.4 ± 5.8 years. Compared with women, men had higher mean BMI, SBP, and DBP. Men also showed higher proportions of overweight/obesity, central obesity, impaired fasting glucose or diabetes, hypertriglyceridemia, current smoking, and regular alcohol consumption. In contrast, reduced HDL-C was common in both sexes. The distribution of occupational categories also differed between men and women. Consistent with the definition-specific prevalence analyses, MetS was more frequent in men according to IDF (22.2% vs 12.6%, *P* = .03), revised NCEP ATP III (26.2% vs 15.3%, *P* = .01), and CDS criteria (14.3% vs 8.5%, *P* = .06; Table 1).

**Table 1:** Baseline characteristics of young Chinese healthcare workers stratified by sex.

Characteristic	Overall (N = 420)	Men (n = 126)	Women (n = 294)	*P* value
Age, yr	32.4 ± 5.8	33.20 ± 5.78	32.06 ± 5.78	.065
BMI, kg/m^2^	23.4 ± 3.1	24.80 ± 2.96	22.80 ± 2.96	<.001
SBP, mm Hg	118.0 ± 12.0	123.00 ± 11.56	115.86 ± 11.56	<.001
DBP, mm Hg	74.0 ± 9.0	77.00 ± 8.79	72.71 ± 8.79	<.001
Nurses, n (%)	209 (49.8)	35 (27.8)	174 (59.2)	<.001
Physicians, n (%)	98 (23.3)	42 (33.3)	56 (19.0)	
Technicians, n (%)	63 (15.0)	28 (22.2)	35 (11.9)	
Administrative/logistic staff, n (%)	50 (11.9)	21 (16.7)	29 (9.9)	
Overweight/obesity (BMI ≥ 25 kg/m^2^), n (%)	120 (28.6)	55 (43.7)	65 (22.1)	<.001
Central obesity (IDF WC cutoffs), n (%)	110 (26.2)	45 (35.7)	65 (22.1)	.004
Impaired fasting glucose or diabetes, n (%)	72 (17.1)	31 (24.6)	41 (13.9)	.008
TG ≥ 1.7 mmol/L, n (%)	97 (23.1)	40 (31.7)	57 (19.4)	.006
Reduced HDL-C, n (%)	126 (30.0)	45 (35.7)	81 (27.6)	.094
Current smoking, n (%)	66 (15.7)	48 (38.1)	18 (6.1)	<.001
Regular alcohol consumption, n (%)	76 (18.1)	42 (33.3)	34 (11.6)	<.001
MetS by IDF, n (%)	65 (15.5)	28 (22.2)	37 (12.6)	.012
MetS by revised NCEP ATP III, n (%)	78 (18.6)	33 (26.2)	45 (15.3)	.009
MetS by CDS, n (%)	43 (10.2)	18 (14.3)	25 (8.5)	.073

BMI = body mass index, CDS = Chinese Diabetes Society, DBP = diastolic blood pressure, HDL-C = high-density lipoprotein cholesterol, IDF = International Diabetes Federation, NCEP ATP III = National Cholesterol Education Program Adult Treatment Panel III, SBP = systolic blood pressure, TG = triglycerides, WC = waist circumference.

### 3.2. Prevalence of metabolic syndrome

The overall prevalence of MetS was 15.5% by IDF criteria, 18.6% by revised NCEP ATP III, and 10.2% by CDS (Table 2). For all 3 definitions, MetS was more common in men than in women, with significant sex differences for IDF (22.2% vs 12.6%, *P* = .03) and NCEP ATP III (26.2% vs 15.3%, *P* = .01), and a borderline difference for CDS (14.3% vs 8.5%, *P* = .06).

**Table 2:** Prevalence of metabolic syndrome according to different diagnostic definitions.

Definition	Overall n (%)	Men n/N (%)	Women n/N (%)	*P* value (men vs women)
IDF	65 (15.5%)	28/126 (22.2%)	37/294 (12.6%)	.03
Revised NCEP ATP III	78 (18.6%)	33/126 (26.2%)	45/294 (15.3%)	.01
CDS	43 (10.2%)	18/126 (14.3%)	25/294 (8.5%)	.06

*P* values refer to comparisons between men and women.

CDS = Chinese Diabetes Society, IDF = International Diabetes Federation, MetS = metabolic syndrome, NCEP ATP III = National Cholesterol Education Program Adult Treatment Panel III.

### 3.3. Agreement and diagnostic performance of MetS definitions

Agreement between IDF and NCEP ATP III was almost perfect (κ = 0.82, 95% CI: 0.76–0.88), whereas agreement between IDF and CDS (κ = 0.64, 95% CI: 0.56–0.72) and between NCEP ATP III and CDS (κ = 0.61, 95% CI: 0.52–0.70) was substantial (Table 3A; Fig. 2).

**Figure 2.**
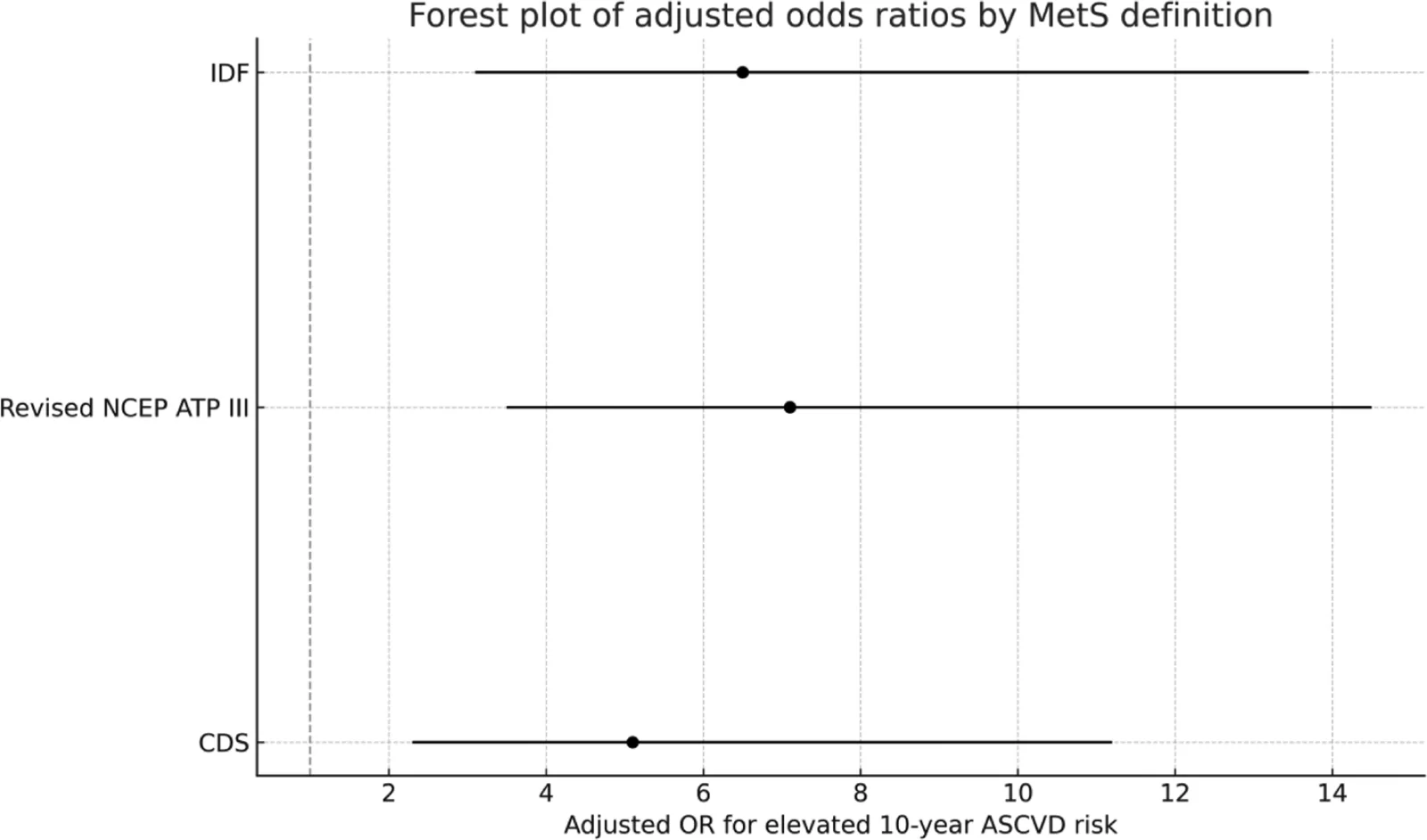
Forest plot of adjusted odds ratios for elevated 10-year ASCVD risk according to different metabolic syndrome definitions. ASCVD = atherosclerotic cardiovascular disease, CDS = Chinese Diabetes Society, NCEP ATP III = National Cholesterol Education Program Adult Treatment Panel III.

CDS demonstrated lower sensitivity (51.3%) but similarly high specificity (99.1%). Its positive and negative predictive values were 93.0% and 89.9%, respectively, with a positive likelihood ratio of 58.5, a negative likelihood ratio of 0.49, a Youden index of 0.50, and a diagnostic odds ratio (OR) of 118.9. The diagnostic AUC was 0.752, with substantial agreement with revised NCEP ATP III (κ = 0.610). Overall, IDF provided a more balanced combination of sensitivity and specificity and showed stronger agreement with revised NCEP ATP III, whereas CDS prioritized specificity at the expense of sensitivity (Table 3B).

### 3.4. Association with and prediction of elevated 10-year ASCVD risk

MetS defined by any of the 3 criteria was strongly associated with elevated 10-year ASCVD risk (China-PAR-estimated risk ≥ 10%; Fig. 3, Table 4). The adjusted ORs for elevated risk were 6.5 (95% CI: 3.1–13.7) for IDF, 7.1 (95% CI: 3.5–14.5) for NCEP ATP III, and 5.1 (95% CI: 2.3–11.2) for CDS (all *P* < .001). All 3 definitions showed good discriminative ability for elevated China-PAR risk (Table 5). The AUCs were 0.82 (95% CI: 0.77–0.87) for NCEP ATP III, 0.80 (95% CI: 0.74–0.85) for IDF, and 0.76 (95% CI: 0.69–0.82) for CDS. Pairwise AUC comparisons using DeLong’s test showed that, compared with CDS, the AUC was significantly higher for NCEP ATP III (*P* = .02) and showed a nonsignificant trend towards higher discrimination for IDF (*P* = .06). Overall, NCEP ATP III and IDF performed similarly and slightly better than CDS in identifying young HCWs at elevated 10-year ASCVD risk (Fig. 3).

**Table 4:** Association between metabolic syndrome and elevated 10-year ASCVD risk (China-PAR-estimated risk ≥ 10%).

Definition	Adjusted OR (95% CI)	*P* value
IDF	6.5 (3.1–13.7)	<.001
Revised NCEP ATP III	7.1 (3.5–14.5)	<.001
CDS	5.1 (2.3–11.2)	<.001

CI = confidence interval, CDS = Chinese Diabetes Society, IDF = International Diabetes Federation, MetS = metabolic syndrome, OR = odds ratio, NCEP ATP III = National Cholesterol Education Program Adult Treatment Panel III.

**Table 5:** Discriminative ability of different metabolic syndrome definitions for elevated 10-year ASCVD risk.

Definition	AUC (95% CI)	*P* vs CDS
IDF	0.80 (0.74–0.85)	.06
Revised NCEP ATP III	0.82 (0.77–0.87)	.02
CDS	0.76 (0.69–0.82)	–

ASCVD = atherosclerotic cardiovascular disease, AUC = area under the curve, CI = confidence interval, CDS = Chinese Diabetes Society, IDF = International Diabetes Federation, MetS = metabolic syndrome, NCEP ATP III = National Cholesterol Education Program Adult Treatment Panel III.

**Figure 3.**
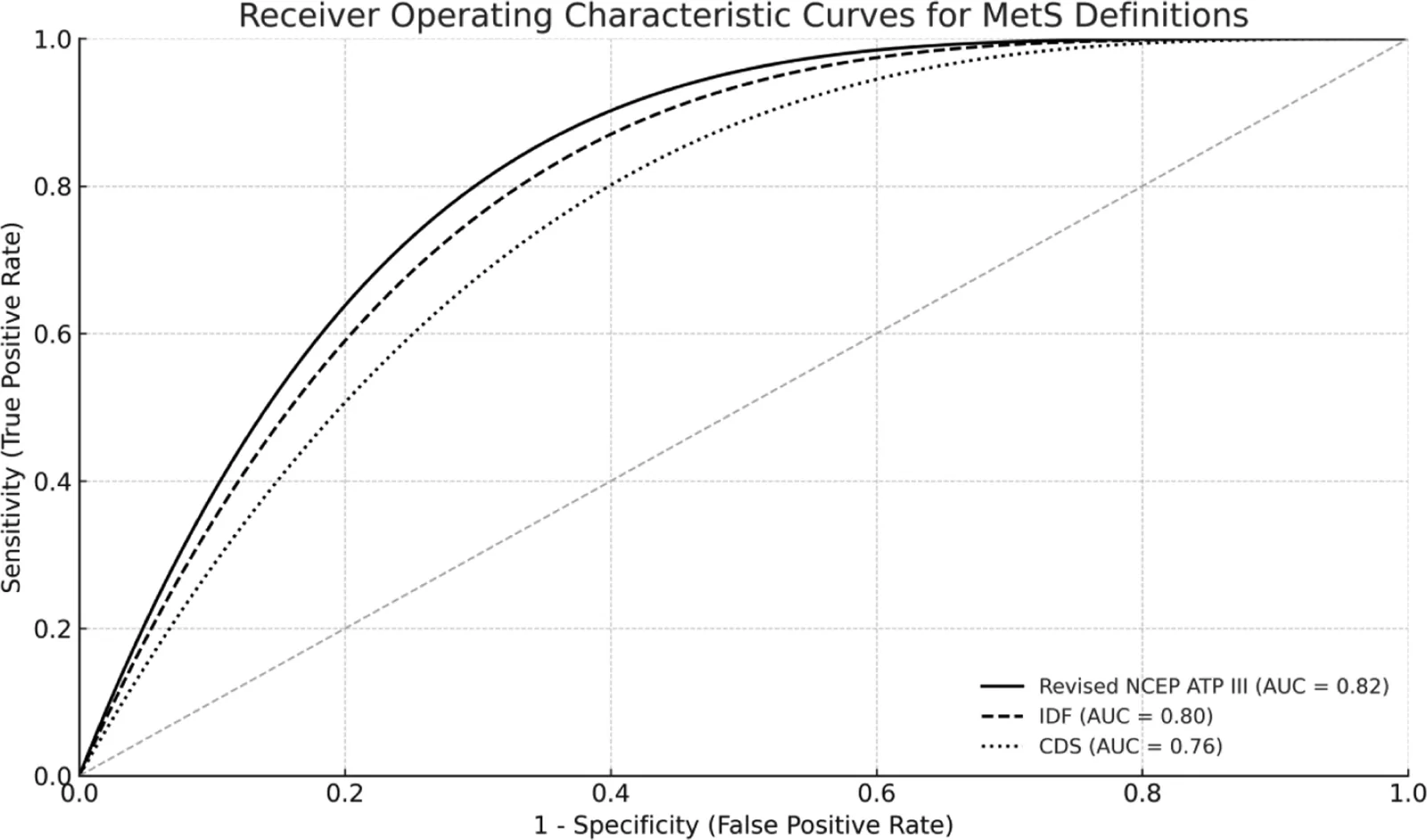
Receiver operating characteristic curves of different metabolic syndrome definitions for predicting elevated 10-year ASCVD risk. ASCVD = atherosclerotic cardiovascular disease, AUC = area under the curve, CDS = Chinese Diabetes Society, IDF = International Diabetes Federation.

Age-range consideration for China-PAR analysis: Among the 420 participants, 344 (81.9%) were younger than 35 years, and 76 (18.1%) were aged 35 to 45 years. Because most participants were below the original validated age range of the China-PAR equations, the ROC and logistic-regression analyses based on China-PAR should be interpreted as exploratory estimated risk stratification rather than definitive prediction of observed 10-year ASCVD events. The small size of the 35 to 45-year subgroup limited the stability and precision of separate age-restricted estimates; therefore, the comparative conclusions were based primarily on the full-sample analysis, with this limitation explicitly considered in the interpretation.

## 4. Discussion

In this retrospective cross-sectional study of young Chinese HCWs, we found that the prevalence of MetS ranged from 10.2% to 18.6%, depending on the diagnostic definition used. Although these estimates are somewhat lower than those reported in many general Chinese adult and community-based cohorts, they still indicate a meaningful cardiometabolic burden in a relatively young and ostensibly healthy hospital workforce.^[12]^ The overall prevalence in our cohort was also slightly lower than that observed in a large employee population in Northeast China, where age-standardized MetS prevalences of 22.9%, 20.6%, and 15.3% were reported for revised NCEP ATP III, IDF, and CDS criteria, respectively, which may reflect our younger age range and a “healthy worker” effect.^[13]^

Consistent with previous studies, MetS was more common in men than in women according to all 3 definitions. This pattern may be partly explained by higher rates of central obesity, smoking, and alcohol use among men, together with sex-specific differences in fat distribution and hormonal milieu. Similar male predominance has been reported in Chinese urban and rural populations and in various occupational groups, suggesting that workplace-based prevention strategies should pay particular attention to younger men.^[12,13]^

A major finding of this study is the systematic difference in MetS prevalence across diagnostic systems, with the revised NCEP ATP III definition yielding the highest prevalence, the IDF definition intermediate, and the CDS definition the lowest. This pattern closely mirrors observations from nationwide Chinese surveys and rural or employee-based cohorts, where ATP III–based criteria generally identify more cases than IDF, while CDS consistently produces the most conservative estimates.^[12-14]^ These discrepancies reflect underlying conceptual and operational differences. NCEP ATP III treats all 5 components as equally weighted and does not require central obesity; the IDF definition mandates central obesity and uses ethnic-specific WCs; and the CDS definition replaces WC with BMI and applies higher thresholds for blood pressure and fasting glucose. In a relatively lean and young hospital workforce, these differences translate into notable variation in both the proportion and the phenotype of individuals classified as having MetS.

Our agreement analyses support this interpretation. The almost perfect concordance between IDF and NCEP ATP III (κ = 0.82) indicates that these 2 definitions identify largely overlapping groups, whereas the only substantial agreement between CDS and the other 2 criteria (κ ≈ 0.6) suggests that CDS selects a narrower subset with more advanced cardiometabolic abnormalities. When we used NCEP ATP III as a pragmatic reference, IDF criteria provided high sensitivity and specificity, whereas CDS prioritized specificity at the expense of sensitivity. Similar trade-offs have been reported in rural and community-based Chinese cohorts, in which CDS definitions tend to identify a smaller, more severely affected subgroup with greater obesity, hypertension, and hyperglycemia.^[14,15]^ From the perspective of occupational screening, IDF or NCEP ATP III therefore appear more suitable when the aim is broad case-finding, whereas CDS may be more appropriate when resources are limited and only the highest-risk individuals can undergo intensive follow-up. The age range of the China-PAR equations also requires careful interpretation. Because 344 participants (81.9%) were younger than 35 years, the full-sample ASCVD risk analyses extend the China-PAR model below its original validated age range. We therefore treated China-PAR results as estimated risk stratification rather than formal long-term risk prediction for the younger subgroup. The subgroup aged 35 to 45 years included only 76 participants (18.1%), which limited the precision of separate age-restricted estimates. Accordingly, the main comparative interpretation focuses on the relative performance of the 3 MetS definitions in the full cohort, while acknowledging that longitudinal validation in appropriately aged populations is needed.

Importantly, all 3 MetS definitions were strongly associated with elevated 10-year ASCVD risk as estimated by the China-PAR equations, with adjusted ORs of approximately 5 to 7. These effect sizes are consistent with previous evidence that MetS is a robust marker of cardiovascular risk in Chinese populations and that different definitions, despite yielding varying prevalence, generally confer similar relative risks for coronary heart disease and stroke.^[16,17]^ Our ROC analyses further showed that the 3 criteria had broadly comparable discriminative ability for identifying individuals with China-PAR risk ≥ 10%, with AUCs between 0.76 and 0.82. NCEP ATP III and IDF performed slightly better than CDS, which is in line with reports that definitions using intermediate thresholds for blood pressure and glucose may capture cardiometabolic risk gradients more effectively than more stringent cutoffs.

### 4.1. Strength

This study also contributes to the relatively limited literature on MetS among HCWs. Studies from Taiwan and other Asian settings have reported MetS prevalences of approximately 8% to 15% in hospital employees, with higher rates in physicians and administrative staff than in nurses and technicians, and strong associations with age, male sex, adiposity, and unhealthy lifestyle factors.^[16-18]^ Moreover, recent work has shown links between MetS, work-related burnout, and psychosocial stress in tertiary hospital staff, suggesting that organizational and psychosocial determinants may play an important role in metabolic health among HCWs.^[18,19]^ Our demonstration of a nontrivial MetS burden in young HCWs, combined with its strong association with elevated China-PAR risk, underscores the need to integrate cardiometabolic risk assessment into routine occupational health programs rather than assuming that medical staff are inherently at low risk. Potential occupational and regional biases should be considered when interpreting the prevalence estimates. The study was conducted in a single tertiary hospital, and the distribution of physicians, nurses, technicians, and administrative/logistic staff may not reflect that of other hospitals or healthcare systems. Work schedules, shift intensity, occupational stress, health awareness, access to medical resources, dietary patterns, and local socioeconomic factors may all influence cardiometabolic risk. In addition, HCWs who remain employed and participate in mandatory annual health examinations may differ from the general working population, raising the possibility of a healthy-worker effect. Therefore, the absolute prevalence estimates should not be directly generalized to all Chinese HCWs or to community populations in other regions, although the comparative pattern across MetS definitions is likely informative for similar hospital-based screening settings.

The use of the China-PAR equations is another strength of our study, as these models were developed and validated in contemporary Chinese cohorts and show good discrimination and calibration for ASCVD prediction across multiple regions and subgroups. Although recent evaluations suggest that China-PAR may underpredict risk in some higher-risk strata and that local recalibration can further improve performance,^[20]^ our goal was not to revalidate the model but to assess how closely simple MetS definitions align with an accepted Chinese risk-prediction tool. In this context, the similar AUCs across definitions suggest that, in young HCWs, the choice between IDF and NCEP ATP III may be driven more by screening philosophy and feasibility than by major differences in risk discrimination.

### 4.2. Limitations

Several limitations should be acknowledged. First, the retrospective cross-sectional design precludes causal inference and does not permit assessment of incident cardiovascular events; the study relies on estimated rather than observed long-term risk. Second, the single-center setting, modest sample size, and restricted age range limit generalizability to other hospitals, regions, and older HCWs. Third, China-PAR was developed for adults aged 35 to 74 years, whereas 344 participants (81.9%) were younger than 35 years. The full-sample China-PAR analyses therefore represent extrapolated risk stratification and should not be interpreted as validated 10-year risk prediction. Fourth, detailed shift schedules, workload intensity, diet, psychosocial stress, objectively measured sleep, and other potentially important occupational exposures were not systematically available, leaving potential residual confounding. Fifth, routine retrospective records may contain measurement or recording error despite source verification. Sixth, NCEP ATP III was used as a pragmatic reference rather than a true diagnostic gold standard. Finally, the Table 3B values in this working draft were internally reconstructed from aggregate case counts and κ values because the participant-level 2 × 2 tables were not available for this revision; they must therefore be verified against the original database before journal submission.

### 4.3. Knowledge gaps

Important knowledge gaps remain. It is unknown which MetS definition best predicts observed long-term cardiovascular and metabolic outcomes in young Chinese HCWs. The clinical significance of discordant classification – particularly participants identified by IDF or NCEP ATP III but not CDS – has not been established. The influence of shift work, night-duty burden, psychosocial stress, sleep disruption, and occupational category on definition-specific MetS risk also remains insufficiently characterized. In addition, validated cardiovascular risk models for Chinese adults younger than 35 years are lacking, limiting robust absolute-risk estimation in the youngest workers.

### 4.4. Future directions

Future research should prioritize multicenter longitudinal cohorts with standardized occupational-exposure measurement and adjudicated cardiometabolic outcomes. Analyses should compare not only prevalence and AUC but also calibration, net reclassification, decision-curve utility, and cost-effectiveness across MetS definitions. Younger-age risk models should be developed or validated for Chinese populations. Randomized or pragmatic workplace trials should then test whether screening linked to targeted interventions – such as physical-activity programs, weight management, shift-schedule optimization, sleep-health strategies, and structured follow-up – improves clinically meaningful outcomes.

## 5. Conclusion

Among 420 young Chinese HCWs in a single tertiary hospital, estimated MetS prevalence differed materially across IDF, revised NCEP ATP III, and CDS criteria and was higher in men. IDF and NCEP ATP III identified more cases and showed stronger agreement with one another than with CDS. All 3 definitions were associated with elevated China-PAR-estimated risk, but these risk analyses are exploratory because most participants were younger than the validated China-PAR age range. The findings support careful selection and transparent reporting of MetS criteria in occupational screening; they do not establish that national diagnostic practice should change. Prospective multicenter studies with observed outcomes are needed before 1 definition can be recommended as clinically superior.

**Table 3A:** Agreement between different diagnostic definitions of metabolic syndrome.

Comparison	Kappa (95% CI)
IDF vs NCEP ATP III	0.82 (0.76–0.88)
IDF vs CDS	0.64 (0.56–0.72)
NCEP ATP III vs CDS	0.61 (0.52–0.70)

Kappa values indicate agreement between 2 definitions beyond chance.

IDF, NCEP ATP III and CDS as defined in Table 2.

CI = confidence interval, CDS = Chinese Diabetes, NCEP ATP III = National Cholesterol Education Program Adult Treatment Panel III.

**Table 3B:** Diagnostic performance of IDF and CDS criteria using revised NCEP ATP III as the pragmatic reference.

Diagnostic index	IDF	CDS
TP/FP/FN/TN	61/4/17/338	40/3/38/339
Sensitivity, %	78.2	51.3
Specificity, %	98.8	99.1
Positive predictive value (PPV), %	93.8	93.0
Negative predictive value (NPV), %	95.2	89.9
Positive likelihood ratio (LR+)	66.9	58.5
Negative likelihood ratio (LR−)	0.22	0.49
Youden index	0.77	0.50
Diagnostic AUC	0.885	0.752
Diagnostic odds ratio (DOR)	303.2	118.9
Cohen κ	0.823	0.610

AUC = area under the curve, CDS = Chinese Diabetes Society, DOR = diagnostic odds ratio, LR+ = positive likelihood ratio, LR− = negative likelihood ratio, MetS = metabolic syndrome, NPV = negative predictive value, PPV = positive predictive value.

## Author contributions

**Conceptualization:** Li Ma, Lei Lei, Zimin Cheng, Yuanyuan Lv, Jie Zheng, Xinyu Chen, Yimeng Huang, Xiaolei Luo, Xiaoliu Li.

**Data curation:** Li Ma, Lei Lei, Zimin Cheng, Yuanyuan Lv, Jie Zheng, Xinyu Chen, Yimeng Huang, Xiaolei Luo, Xiaoliu Li.

**Formal analysis:** Li Ma, Lei Lei, Zimin Cheng, Yuanyuan Lv, Jie Zheng, Xinyu Chen, Yimeng Huang, Xiaolei Luo, Xiaoliu Li.

**Funding acquisition:** Li Ma, Lei Lei, Zimin Cheng, Yuanyuan Lv, Jie Zheng, Xinyu Chen, Yimeng Huang, Xiaoliu Li.

**Investigation:** Li Ma, Xiaoliu Li.

**Writing – original draft:** Xiaoliu Li.

**Writing – review & editing:** Xiaoliu Li.
